# Mental health disorders and utilization of mental healthcare services in United Nations personnel

**DOI:** 10.1017/gmh.2019.29

**Published:** 2020-02-12

**Authors:** Adam D. Brown, Katharina Schultebraucks, Meng Qian, Meng Li, Danny Horesh, Carol Siegel, Yosef Brody, Abdalla Mansour Amer, Rony Kapel Lev-Ari, Francis Mas, Charles R. Marmar, Jillann Farmer

**Affiliations:** 1 Department of Psychiatry, New York University School of Medicine, 1 Park Avenue, 8th Floor, New York, NY 10016, USA; 2 Department of Psychology, New School for Social Research, 80 5th Avenue, 6th Floor, New York, NY 10003, USA; 3 Department of Psychology, Bar-Ilan University, Ramat-Gan 52900, Tel-Aviv, Israel; 4 United Nations Healthcare Management and Occupational Safety and Health Division, United Nations Secretariat, Secretariat Building, Room S-0544, 405 East 42nd Street, New York, NY 10017, USA; 5 Critical Incident Stress Management Unit, United Nations Department of Safety and Security, New York, NY 10017, USA

**Keywords:** Human rights, secondary trauma, trauma, United Nations

## Abstract

**Background:**

United Nations (UN) personnel address a diverse range of political, social, and cultural crises throughout the world. Compared with other occupations routinely exposed to traumatic stress, there remains a paucity of research on mental health disorders and access to mental healthcare in this population. To fill this gap, personnel from UN agencies were surveyed for mental health disorders and mental healthcare utilization.

**Methods:**

UN personnel (*N* = 17 363) from 11 UN entities completed online measures of generalized anxiety disorder (GAD), major depressive disorder (MDD), posttraumatic stress disorder (PTSD), trauma exposure, mental healthcare usage, and socio-demographic information.

**Results:**

Exposure to one or more traumatic events was reported by 36.2% of survey responders. Additionally, 17.9% screened positive for GAD, 22.8% for MDD, and 19.9% for PTSD. Employing multivariable logistic regressions, low job satisfaction, younger age (<35 years of age), greater length of employment, and trauma exposure on or off-duty was significantly associated with all the three disorders. Among individuals screening positive for a mental health disorder, 2.05% sought mental health treatment within and 10.01% outside the UN in the past year.

**Conclusions:**

UN personnel appear to be at high risk for trauma exposure and screening positive for a mental health disorder, yet a small percentage screening positive for mental health disorders sought treatment. Despite the mental health gaps observed in this study, additional research is needed, as these data reflect a large sample of convenience and it cannot be determined if the findings are representative of the UN.

## Introduction

United Nations (UN) personnel seek to address a wide range of political, social, environmental, and cultural crises throughout the world (e.g. UN Department of Economic and Social Affairs (n.d.)). Although the focus of UN work varies widely across entities and job type, UN personnel may be at high risk for psychiatric disorders as their work may involve exposure to high levels of stress and trauma. For example, several UN entities are directly involved in providing humanitarian relief in response to war, environmental disasters, and other emergencies (United Nations Disaster Assessment and Coordination. UNDAC Field Handbook, 2013). UN personnel may also be exposed to considerable stress and trauma through human rights policy and advocacy work, which often entails the documentation human rights violations through interviewing victims, reviewing evidence, and visiting physical sites of human rights violations (Gallagher, 2008). Moreover, the UN also employs sanctioned peacekeepers – military personnel under the UN command – involved in monitoring and observing peace processes in post-conflict settings and providing security in regions where there are current conflicts (Ratner, 1995). Although the primary objectives of UN personnel may vary considerably, the stressors and challenges they face may overlap considerably as they often work in the similar contexts, engage in each other's roles (e.g. advocates and peacekeepers may help deliver aid), and influence one another's work.

Although no studies to our knowledge have been conducted with UN personnel, a growing body of research suggests that individuals engaged in humanitarian aid, human rights advocacy, and peacekeeping report high levels of direct and secondary trauma. The International Committee of the Red Cross (ICRC) recently reported that over the past 2 years there have been approximately 1200 incidents of violence against health-care workers in 16 countries (ICRC, 2018). There is also a growing body of evidence documenting risks associated with human rights advocacy and activism. Amnesty International recently launched a campaign, ‘Brave. Human Rights Defenders Under Threat: A Shrinking Space for Civil Society’ documenting an increase in death threats against human rights advocates^6^ and advocates report high levels of direct and secondary trauma (Amnesty International, 2017). Peacekeepers are also exposed to direct threat and violence. A recent study found that a majority of Australian peacekeepers reported being in a situation in which there was a threat of injury (83%) or death [73%, (Forbes *et al*., 2016))]. In a 2015 report, the High-Level Independent Panel on UN Peace Operations emphasized that UN personnel operate in ‘increasingly dangerous environments’ (Willmot *et al*., 2015).

Compared to other populations routinely exposed to traumatic stress in the context of their work, such as combat veterans, there remains a paucity of research on psychiatric morbidity associated with individuals involved in aid work, peacekeeping, and human rights advocacy. To date, studies investigating rates of psychiatric disorders in humanitarian aid workers vary considerably from 8 to 43% for PTSD and 8–20% for depression (Connorton *et al*., 2012). Only two studies have been conducted with human rights advocates in which rates of depression ranged from 8.6 to 16% and PTSD from 7.1 to 20% (Holtz *et al*., 2002; Joscelyne *et al*., 2015). A meta-analysis of peacekeeper studies reported that rates of PTSD ranged from 0.05 to 2.8% [pooled prevalence rates = 5.3% (Souza *et al*., 2011)].

The extant data are based primarily on studies with small samples, in specific contexts, employing different methods of assessment and as such, it is difficult to determine the levels of psychiatric disorders in this population. Furthermore, even less is known about the occupational and organizational factors that might be associated with psychiatric vulnerability. Clarifying those factors associated with risk for psychiatric disorders could serve as an important guide for policy-makers to prevent and reduce the psychiatric burden on this global workforce. Furthermore, little is known about the potential barriers to accessing mental healthcare in this population. To fill these gaps, UN personnel were asked to complete an online survey screening for psychiatric disorders, socio-demographic questions, and utilization of mental healthcare services.

## Methods

### Participants

Organizations which had a direct service–delivery relationship with the Medical Services Division of the UN were invited to participate. This included the New York-based agencies, funds and programs, and the various UN secretariat entities around the world, including peacekeeping missions, Economic Commissions and the UN offices in Geneva, Nairobi, and Vienna. Specifically, the participating entities included Department of Field Support (DFS), United Nations Development Programme (UNDP), United Nations Office at Nairobi (UNON), the United Nations Children's Fund (UNICEF), Secretariat Headquarters in NY, United Nations Department of Safety and Security (UNDSS), United Nations Office at Geneva (UNOG), Economic Commission for Latin America and the Caribbean (ECLAC), Economic and Social Commission for Asia and the Pacific (ESCAP), United Nations Office at Vienna (UNOV), and Economic and Social Commission for Western Asia (ESCWA).

A total of 45 544 participants from 11 UN entities were invited to participate in the study.^1^^†^ In total, 17 363 UN personnel participated in the survey, representing a convenience sample of 32.8% personnel employed by participating agencies and 17.7% of all UN personnel (see Table 1 for demographic and occupation-related sample characteristics of survey respondents). Although our sample differed from available UN-wide demographic data on Gender, Age, Parental Status, Contract Type, and Duty Station Type (*p* < 0.0001), Cramer's *V* showed that the effect sizes were small (*φ*c =  0.07–0.14).

**Table 1.** Demographic and occupational characteristics of UN staff members (*n*  =  17 363)

	Sample characteristics	Global staff population	Significance	Craver's *V*
Gender				
Female	9106 (53%)	42 123 (43%)	χ^2^(1) = 592.62, *p* < 2.2 × 10^−16^	0.0717
Male	8103 (47%)	56 032 (57%)		
Missing data	154			
Age bracket				
34 and Below	3540 (20%)	12 637 (13%)	χ^2^(5) = 743.3, *p* < 2.2 × 10^−16^	0.0797
35–39	3376 (20%)	16 881 (17%)		
40–44	3186 (18%)	18 756 (19%)		
45–49	2672 (15%)	17 601 (18%)		
50–54	2418 (14%)	16 012 (16%)		
55-Above	3540 (12%)	16 268 (17%)		
Missing data	78			
Relationship status				
Not in a relationship	3851 (22%)	N/A		
In a partnership recognized by the UN	11 177 (66%)	N/A		
In a partnership not recognized by the UN	2043 (12%)	N/A		
Missing data	292			
Dependent child				
Yes	10 809 (63%)	43 323 (44%)	χ^2^(1) = 2022.7, *p* < 2.2 × 10^−16^	0.1324
No	6438 (37%)	54 832 (56%)		
Missing data	116			
Type of appointment				
Permanent/continuous	3502 (24%)	26 567 (27%)	χ^2^(2) = 2350.5, *p* < 2.2 × 10^−16^	0.1446
Fixed term	9219 (64%)	68 879 (70%)		
Temporary	1584 (11%)	2709 (3%)		
Consultant	152 (1%)	N/A		
Missing data	2906			
Type of duty station				
Family duty station	9300 (79%)	N/A		
Non-family duty station	2407 (21%)	N/A		
Missing data	715			
Length of employment within UN				
Less than 1 year	2387 (15%)	N/A		
1–3 years	2821 (18%)	N/A		
3–5 years	2189 (14%)	N/A		
5–10 years	4038 (25%)	N/A		
More than 10 years	4594 (28%)	N/A		
Missing data	1334			

*Note*: count (percentage).

Among those who participated in the survey, 79.27% (*N* = 13 763) completed all of the questions. A series of χ^2^ tests were conducted comparing the demographic variables among survey responders that completed all three clinical measures, partially completed the clinical measures, and did not complete any of the clinical measures (see supplemental materials for complete analyses). No differences were found between those who completed *v.* those who partially completed the clinical self-report measures on Gender, Age, Relationship Status, Duty Station Type, and Duration of Employment for the UN. A marginally significant difference was found for Contract Type. Those who completed the clinical measures were more likely to be on fixed-term appointments (χ^2^ = 3.84, *p* = 0.05) whereas those who partially completed the clinical measures were more likely to be on temporary appointment (χ^2^ = 4.00, *p* = 0.05). When comparing those who completed the clinical measures, compared to those that did not complete any of the clinical measures, there were no differences found for gender or duty station type. Individuals that did not complete the surveys were more likely to be below 34 and younger (χ^2^ = 72.95, *p* < 0.0001), not in a relationship (χ^2^ = 17.00, *p* < 0.0001), worked for the UN for <1 year (χ^2^ = 18.48, *p* < 0.0001) or between 1 and 3 years (χ^2^ = 4.43, *p* < 0.05), and were on temporary (χ^2^ = 16.25, *p* < 0.05) or consultancy appointments (χ^2^ = 3.87, *p* < 0.05).

All UN personnel from these 11 UN entities were eligible for inclusion in the study. Missing data were accommodated using case-wise deletion. The data for this cross-sectional prevalence study was originally collected as part of an internal assessment carried out by the Medical Services Division of the UN. The de-identified data were shared with the NYU School of Medicine for analysis. The NYU School of Medicine Institutional Review Board determined that the analysis of these de-identified archival data was exempt from IRB review per 45 CFR 46.101(b) #4.

### Procedures

The survey was distributed by the UN Medical Service Division to UN personnel at the 11 UN agencies over an 8 month period between 2015 and 2016. Data were collected via an on-line questionnaire using SurveyMonkey. An e-mail with information on the purpose of the survey, confidentiality clauses, the approximate time needed to complete the survey, and a link to the survey was sent to the personnel of all participating organizations. No personally identifying information was collected.

### Measures

#### Demographics

Individuals were asked to complete a series of demographic questions about gender, age, relationship status, parental status, and duration of UN employment. Participants were also asked about the type of appointment (e.g. permanent, temporary, consultant), recruitment type (local *v.* international), and duty station type (family *v.* non-family). Participation in the survey was voluntary. The survey was available primarily in English for the majority of the organizations.^2^

#### Trauma exposure

Participants were asked if they had experienced or witnessed an event in the past 12 months that involved actual or threatened death or serious injury either during work or while they were off-duty.

#### Generalized anxiety disorder (GAD)

Gad was measured with the 7-item self-report instrument GAD-7 (Spitzer *et al*., 2006). The GAD-7 assesses symptoms of GAD within the past 2 weeks. A cut-off score of ⩾10 is defined as a positive screen for GAD (Spitzer *et al*., 2006).

#### Major depressive disorder (MDD)

MDD was screened with the Patient Health Questionnaire-9 (PHQ-9), which is a validated 9-item self-report screening instrument for depression (Kroenke *et al*., 2001). The PHQ-9 assesses symptoms of depression over the past 2 weeks. A cut-off score of ⩾10 defines a positive screen for MDD (Kroenke *et al*., 2001).

#### Posttraumatic stress disorder (PTSD)

To screen for PTSD, the 6-item PTSD Checklist-6 (PCL-6) was used (Lang *et al*., 2012). The PCL assesses symptoms of PTSD within the past month. A cut-off score of ⩾14 is considered a positive screen for PTSD in the PCL-6 (Lang *et al*., 2012).

#### Mental healthcare utilization

Participants were asked if they had felt the need to see a mental health professional (e.g. staff counselor) within the past 12 months and if they were currently receiving mental health services within or outside the UN.

### Statistical analysis

**Fig. 1.**
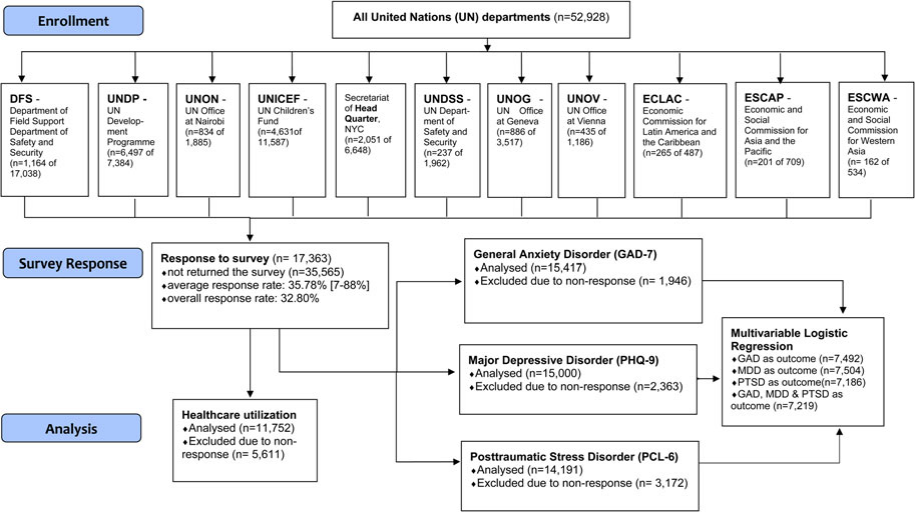
Flow diagram: United Nations Mental Well-Being Survey.

Estimated point prevalence rates of participants who screened positive for GAD, MDD, or PTSD were ascertained as were the rates of reported mental healthcare utilization in the past 12-months. Multivariable logistic regression analyses were used to examine the associations among demographic as well as occupational factors and GAD, MDD, or PTSD. The binary outcome variable was case status for each participant with separate models run for each of the three disorders. The statistical significance level employed in the models was *α* = 0.05. The preparation of the paper followed the statement for strengthening the reporting of observational studies in epidemiology [STROBE (Vandenbrouckel *et al*., 2007; von Elm *et al*., 2007) (see Figure 1)].

## Results

### Estimated point prevalence of screen positives for GAD, MDD, and PTSD

**Fig. 2.**
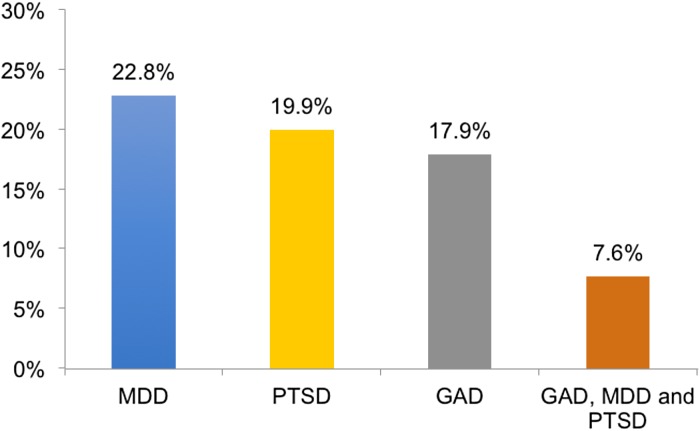
Percentages of UN employees who screen positive for mental health disorders.

*Note*. MDD, major depressive disorder; PTSD, posttraumatic stress disorder; GAD, generalized anxiety disorder. The percentages refer to complete-case-analysis of the screening instruments for GAD (*n*  =  15 417), PTSD (*n*  =  14 191), MDD (*n*  =  15 000) and GAD, MDD and PTSD together (*n*  =  13 958).

Among survey respondents, 9118 individuals (65.32%) did not meet the screening criteria for any for the measured mental health disorders. However, 17.9% (*N* = 2759) reported symptoms that met the threshold for GAD, 22.8% (*N* = 3417) for MDD, and 19.9% (*n* = 2823) for PTSD (see Fig. 2). In addition, 34.67% (*n*  =  4840) of UN members screened positive for at least one mental health disorder, 18.01% (*n*  =  2523) screened positive for at least two mental health disorders, and 7.62% (*n*  =  1064) screened positive for all three mental health disorders.

### Predictors of mental health disorders

To identify predictors of GAD, MDD, and PTSD a series of multivariable logistic regressions were conducted (see Tables 1–4).

#### Generalized anxiety disorder

The parameter estimates of multivariable logistic regressions are reported in Table 2. When accounting for all predictors in the multivariable logistic regression, several factors remained statistically significant as predictors of GAD (see Table 2). GAD was significantly associated with low job satisfaction (*p* < 0.0001), followed by longer duration of UN employment (*p* < 0.0001), younger age (*p* < 0.001), and trauma exposure on (*p* < 0.001) or off-duty (*p* < 0.0001).

**Table 2.** Parameter estimates for the multivariable logistic regression including covariates to predict the presence of generalized anxiety disorder in UN staff members (*n*  =  12 732)

Variable	*B*	s.e.	*p*	OR	95% CI	
Intercept	−1.0401	0.1665	<0.0001			
Age (categories below *v.* age 34 and below)						
Age: 35–39	0.132	0.069	0.0558	0.819	0.659	1.017
Age: 40–44	−0.0661	0.0691	0.3386	0.672	0.535	0.844
Age: 45–49	−0.0306	0.0745	0.6817	0.696	0.546	0.888
Age: 50–54	−0.2512	0.0809	**0.0019**	0.558	0.432	0.722
Age: 55 and above	−0.116	0.0844	0.1694	0.639	0.491	0.831
Gender (category below *v.* female gender)						
Male	−0.0623	0.0345	0.0711	0.883	0.771	1.011
Relationship status (categories below *v.* not in a relationship)						
In a partnership not recognized by your organization	0.0246	0.0686	0.7194	1.001	0.797	1.258
In a partnership recognized by your organization	−0.0479	0.0531	0.3675	0.931	0.783	1.108
Parenthood status (category below *v.* no dependent child)						
Dependent children	−0.0322	0.0392	0.4115	0.938	0.804	1.093
Duration of employment (categories below *v.* <1 year)						
Years worked at UN: 1–3 Years	−0.0339	0.0905	0.7082	1.941	1.386	2.719
Years worked at UN: 3–5 Years	−0.0136	0.0876	0.8762	1.981	1.411	2.783
Years worked at UN: 5–10 Years	0.282	0.0633	**<0.0001**	2.663	1.958	3.621
Years worked at UN: more than 10 Years	0.4629	0.0738	**<0.0001**	3.191	2.307	4.413
Type of appointment (categories below *v.* permanent)						
Consultant	0.7481	0.4633	0.1063	2.914	0.863	9.842
Fixed term	−0.151	0.1612	0.3489	1.186	0.997	1.411
Temporary	−0.2757	0.1779	0.1211	1.047	0.79	1.388
Recruitment type (categories below *v.* local)						
International	0.0105	0.0324	0.7453	1.021	0.9	1.159
Job satisfaction (categories below *v.* not at all satisfied)						
Extremely satisfied	−0.9334	0.1417	**<0.0001**	0.1	0.067	0.151
Very satisfied	−1.0183	0.0699	**<0.0001**	0.092	0.071	0.12
Moderately satisfied	−0.1092	0.0597	0.0671	0.228	0.178	0.292
Slightly satisfied	0.6936	0.0774	**<0.0001**	0.51	0.387	0.672
Trauma exposure (categories below *v.* trauma exposure during last 12 months)						
No trauma exposure: work	−0.242	0.0437	**<0.0001**	0.616	0.519	0.732
No trauma exposure: home	−0.2618	0.0417	**<0.0001**	0.592	0.503	0.698

*Note*: AUC = 0.73. Values in bold text connote statistical significance, *p* < 0.05, *p* < 0.01, *p* < 0.001, *p* < 0.0001.

#### Major depressive disorder

Similarly, multivariable logistic regression revealed that MDD associated with low job satisfaction (*p* < 0.0001), followed by longer duration of UN employment (*p* < 0.0001), younger age (*p* < 0.01), and trauma exposure on (*p* < 0.01) or off duty exposure (*p* < 0.01), (see Table 3).

**Table 3.** Parameter estimates for the multivariable logistic regression including covariates to predict the presence of major depressive disorder in UN staff members (*n*  =  12 732)

Variable	*B*	s.e.	*p*	OR	95% CI	
Intercept	−1.0594	0.1675	<0.0001			
Age (categories below *v.* age 34 and below)						
Age: 35–39	0.0314	0.073	0.6674	0.758	0.604	0.951
Age: 40–44	0.0548	0.07	0.4336	0.776	0.614	0.98
Age: 45–49	0.0516	0.0759	0.4962	0.773	0.603	0.993
Age: 50–54	−0.2135	0.0829	**0.01**	0.593	0.455	0.773
Age: 55 and above	−0.233	0.0893	**0.0091**	0.582	0.442	0.767
Gender (category below *v.* female gender)						
Gender	−0.0376	0.0356	0.2918	0.928	0.807	1.067
Relationship status (categories below *v.* not in a relationship)						
Relationship status: in a partnership not recognized by your organization	−0.0177	0.0709	0.8023	0.868	0.687	1.096
In a partnership recognized by your organization	−0.1061	0.0549	0.053	0.795	0.666	0.948
Parenthood status (category below *v.* no dependent child)						
Dependent children	−0.0223	0.0405	0.5823	0.956	0.816	1.121
Duration of employment (categories below *v.* <1 year)						
Years worked at UN: 1–3 years	−0.2777	0.0982	**0.0047**	1.149	0.821	1.606
Years worked at UN: 3–5 years	0.0447	0.0887	0.6146	1.586	1.143	2.199
Years worked at UN: 5–10 years	0.2117	0.0652	**0.0012**	1.874	1.396	2.515
Years worked at UN: more than 10 years	0.4377	0.0755	**<0.0001**	2.349	1.719	3.21
Type of appointment (categories below *v.* permanent)						
Consultant	0.7777	0.4651	0.0945	2.955	0.87	10.032
Fixed term	−0.2158	0.1621	0.1831	1.094	0.916	1.307
Temporary	−0.256	0.1788	0.1522	1.051	0.79	1.399
Recruitment type (categories below *v.* local)						
International	0.0321	0.0334	0.3367	1.066	0.935	1.216
Job satisfaction (categories below *v.* not at all satisfied)						
Extremely satisfied	−0.9508	0.1485	**<0.0001**	0.098	0.064	0.149
Very satisfied	−1.0631	0.0735	**<0.0001**	0.087	0.067	0.114
Moderately satisfied	−0.0992	0.0616	0.107	0.229	0.179	0.293
Slightly satisfied	0.7388	0.0787	**<0.0001**	0.53	0.402	0.698
Trauma exposure (categories below *v.* trauma exposure during last 12 months)						
No trauma exposure: work	−0.2704	0.0448	**<0.0001**	0.582	0.488	0.694
No trauma exposure: home	−0.2442	0.043	**<0.0001**	0.614	0.519	0.726

*Note*: AUC = 0.74. Values in bold text connote statistical significance, *p* < 0.05, *p* < 0.01, *p* < 0.001, *p* < 0.0001.

#### Posttraumatic stress disorder

When accounting for all covariates in the multivariable logistic regression analysis, six factors remained statistically significant as predictors of PTSD (see Table 4). These factors were younger age (*p* < 0.0001), not being in a relationship (*p* < 0.05), parents with a dependent child (*p* < 0.05), longer duration of UN employment (*p* < 0.0001), lower job satisfaction (*p* < 0.0001), and trauma exposure during work (*p* < 0.0001) and off-duty (*p* < 0.0001).

**Table 4.** Parameter estimates for the multivariable logistic regression including covariates to predict the presence of posttraumatic stress disorder in UN staff members (*n*  =  12 732)

Variable	*B*	s.e.	*p*	OR	95% CI	
Intercept	−1.0318	0.212	<0.0001			
Age (categories below *v.* age 34 and below)						
Age: 35–39	0.0581	0.0774	0.4534	0.826	0.646	1.057
Age: 40–44	0.1419	0.0726	0.0508	0.898	0.7	1.153
Age: 45–49	0.0586	0.0792	0.4593	0.827	0.633	1.08
Age: 50–54	−0.1018	0.0838	0.2243	0.704	0.534	0.929
Age: 55 and above	−0.4057	0.0961	**<0.0001**	0.52	0.385	0.701
Gender (category below *v.* female gender)						
Male	−0.072	0.0377	0.0563	0.866	0.747	1.004
Relationship status (categories below *v.* not in a relationship)						
Relationship status: In a Partnership not Recognized by your organization	0.0139	0.0754	0.8534	0.887	0.692	1.138
In a partnership Recognized by your organization	−0.1475	0.0583	**0.0114**	0.755	0.625	0.911
Parenthood status (category below *v.* no dependent child)						
Dependent Children	−0.1004	0.0434	**0.0207**	0.818	0.69	0.97
Duration of employment (categories below *v.* <1 year)						
Years worked at UN: 1–3 Years	−0.197	0.1023	0.0541	1.327	0.924	1.905
Years worked at UN: 3–5 Years	0.0625	0.0946	0.5089	1.72	1.202	2.46
Years worked at UN: 5–10 Years	0.1952	0.0695	**0.005**	1.964	1.421	2.715
Years worked at UN: More than 10 Years	0.4191	0.0796	**<0.0001**	2.457	1.747	3.455
Type of appointment (categories below *v.* permanent)						
Consultant	−0.0208	0.6041	0.9725	0.924	0.19	4.502
Fixed term	−0.0469	0.2076	0.8213	0.9	0.748	1.084
Temporary	0.00968	0.2231	0.9654	0.953	0.703	1.291
Recruitment type (categories below *v.* local)						
International	−0.056	0.0352	0.112	0.894	0.779	1.026
Job satisfaction (categories below *v.* not at all satisfied)						
Extremely satisfied	−0.9068	0.1567	**<0.0001**	0.17	0.108	0.269
Very satisfied	−0.5924	0.0728	**<0.0001**	0.233	0.173	0.313
Moderately satisfied	0.0286	0.0666	0.6677	0.434	0.326	0.577
Slightly satisfied	0.6063	0.0895	**<0.0001**	0.772	0.56	1.066
Trauma exposure (categories below *v.* trauma exposure during last 12 months)						
No trauma exposure: Work	−0.5719	0.0424	**<0.0001**	0.319	0.27	0.376
No trauma exposure: Home	−0.7194	0.0397	**<0.0001**	0.237	0.203	0.277

*Note*: AUC = 0.79. Values in bold text connote statistical significance, *p* < 0.05, *p* < 0.01, *p* < 0.001, *p* < 0.0001.

#### Predictors of comorbidity: combination of GAD, MDD, and PTSD

In an attempt to identify the most distressed population of UN employees, multivariable logistic regression was conducted to identify predictors of triple comorbidity (GAD, MDD, and PTSD). After accounting for all covariates in the model, younger age (*p* < 0.0001), female gender (*p* < 0.01), partnership status (single) (*p* < 0.0001), parents with a dependent child (*p* < 0.0001), longer duration of employment with the UN (*p* < 0.0001), lower job satisfaction (*p* < 0.0001), and trauma exposure on (*p* < 0.0001) and off duty (*p* < 0.0001) predicted the group that met current screening criteria for all three disorders (see Table 5).

**Table 5.** Parameter estimates for the multivariable logistic regression including covariates to predict comorbidity of generalized anxiety disorder, major depressive disorder and posttraumatic stress disorder UN staff members (*n*  =  12 732)

Variable	*B*	s.e.	*p*	OR	95% CI	
Intercept	−0.2614	0.1622	0.107			
Age (categories below *v.* Age 34 and below)						
Age: 35–39	0.1115	0.0611	0.068	0.79	0.652	0.957
Age: 40–44	0.000939	0.0594	0.9874	0.707	0.58	0.862
Age: 45–49	0.0247	0.0643	0.7007	0.724	0.586	0.895
Age: 50–54	−0.1998	0.0686	**0.0036**	0.578	0.463	0.722
Age: 55 and above	−0.2851	0.0746	**0.0001**	0.531	0.421	0.67
Gender (category below *v.* female gender)						
Male	−0.1071	0.03	**0.0004**	0.807	0.718	0.908
Relationship status (categories below *v.* not in a relationship)						
Relationship status: in a partnership not recognized by your organization	0.0389	0.0605	0.5202	0.953	0.78	1.164
In a partnership recognized by your organization	−0.1259	0.0464	**0.0067**	0.808	0.695	0.94
Parenthood status (category below *v.* no dependent child)						
Dependent children	−0.0873	0.0343	**0.011**	0.84	0.734	0.961
Duration of employment (categories below *v.* <1 year)						
Years worked at UN: 1–3 years	−0.1389	0.0777	0.074	1.558	1.19	2.039
Years worked at UN: 3–5 years	0.0177	0.0743	0.8114	1.822	1.39	2.387
Years worked at UN: 5–10 years	0.2516	0.0545	**<0.0001**	2.301	1.806	2.933
Years worked at UN: more than 10 years	0.4516	0.0635	**<0.0001**	2.811	2.171	3.639
Type of appointment (categories below *v.* permanent)						
Consultant	0.0942	0.4579	0.837	1.185	0.356	3.94
Fixed term	−0.00982	0.1578	0.9504	1.068	0.919	1.241
Temporary	−0.00902	0.1701	0.9577	1.069	0.84	1.36
Recruitment type (categories below *v.* local)						
International	−0.0261	0.0281	0.3531	0.949	0.85	1.06
Job satisfaction (categories below *v.* not at all satisfied)						
Extremely satisfied	−1.1367	0.1224	**<0.0001**	0.095	0.065	0.139
Very satisfied	−0.8857	0.0588	**<0.0001**	0.122	0.094	0.159
Moderately satisfied	0.022	0.0539	0.6829	0.303	0.235	0.392
Slightly satisfied	0.785	0.0751	**<0.0001**	0.65	0.487	0.868
Trauma exposure (categories below *v.* trauma exposure during last 12 months)						
No trauma exposure: work	−0.356	0.0389	**<0.0001**	0.491	0.421	0.572
No trauma exposure: home	−0.5159	0.0366	**<0.0001**	0.356	0.309	0.411

*Note*: AUC = 0.75. Values in bold text connote statistical significance, *p* < 0.05, *p* < 0.01, *p* < 0.001, *p* < 0.0001.

### Mental healthcare services

Next, the self-reported frequency of mental health services utilization was examined. In the past year 1.68% (*n* = 197) of the total UN personnel in the survey reported receiving mental health services within the UN and 4.58% (*n* = 538) sought mental healthcare services outside the UN. Among those who screened positive for one or more of GAD, MDD, or PTSD, 10.01% reported using current mental health treatment outside of the UN and 2.08% within the UN system. The mental healthcare rate of utilization for individuals that screen positive for GAD, MDD, and PTSD was 7.6%.

Within the past year, 93.75% (*n* = 11 017) of the UN personnel in the total sample reported that they did not receive mental healthcare during the previous year. Among this group, 15.1% (*n* = 858) responded that they did not do so because they were ‘not comfortable’ and 10.2% (*n* = 582) stated that they did not know if such services were available.

## Discussion

This survey found that a high number of UN personnel in this study screened positive for mental health disorders. The percentage of those screening positive for GAD, MDD, or PTSD is considerably higher than the prevalence in the world general population (e.g. Kessler *et al*., 2005). Remarkably, about one-third of all UN personnel surveyed reported symptoms that qualify for screening positive for at least one mental health disorder. This rate is in the upper range of individuals screening positive for anxiety disorders, MDD or PTSD found in personnel that are recurrently exposed to occupational and potentially traumatic stress, such as combat veterans and police (Hoge *et al*., 2004; Marmar *et al*., 2006).

Relative to the high percentage of those screening positive for mental health disorders in this study, UN personnel reported low levels of mental healthcare utilization. These findings suggest that there is a large treatment gap for UN personnel and further underscore the large treatment gaps that have been identified globally (Patel and Prince, 2010). These data are also consistent with unpublished data from UNHCR (Dubravka *et al*., 2016) in which staff reported ‘not feeling comfortable’ with mental health services or not knowing if services were ‘available’. The treatment gap for this population is probably even larger given that all of the individuals in this survey were employed by the UN, an intergovernmental organization that has greater resources than many smaller non-governmental organizations (NGOs) and local agencies throughout the world. Our findings suggest that stigma about seeking mental health services may be a key barrier to care and that organizations in this field would benefit from studying negative attitudes towards help seeking within their staff and incorporating established methods for reducing mental health gaps in occupational settings (Joyce *et al*., 2016).

In addition, a number of factors were significantly associated with GAD, MDD, and PTSD in UN personnel. A consistent factor across all three psychiatric symptom categories was low levels of job satisfaction. Although it cannot be determined from this survey whether low job satisfaction is a risk factor or an outcome of mental health disorders, other studies have found that job satisfaction is a robust predictor of mental health outcomes (Faragher *et al*., 2005) and the strong association between job satisfaction and mental health underscores the need for aid and advocacy groups to clarify which factors impact job satisfaction within their organizations.

In addition, after working for 5 years for the UN, the likelihood of screening positive for a mental health disorder is two times higher than working for <1 year and after 10 years, approximately three times higher than working for <1 year.

The link between duration of employment and risk for psychiatric disorders may be due to cumulative exposure to stress and trauma during and outside of work, which previous research has found to be associated with negative mental health outcomes, such as PTSD and depression (Kraaij and de Wilde, 2001; Breslau *et al*., 2008). Trainings and the identification of mental health vulnerabilities within the first year of work may represent a critical window for preventing and reducing mental health issues within aid and advocacy organizations. However, given the cross-sectional nature of this study, prospective longitudinal studies are warranted to further shed light on these findings.

Another significant predictor for psychiatric disorders was the exposure to a traumatic event within the past 12 months as reported by UN personnel, either during work or while off-duty. These findings suggest that given the high rates of trauma exposure among UN personnel and its relation to negative mental health outcomes, mental health programs within the UN can target those staff whose jobs will most likely involve direct or indirect exposure. Future research would benefit from testing whether resilience training programs or mental health screening initiatives employed by other fields in which personnel are often exposed to trauma could be adapted for this population (Adler *et al*., 2009; McCraty and Atkinson, 2012).

Although it might be expected that UN staff would be exposed to high levels of trauma during work, rates of exposure were also high when UN staff were off-duty. Additional follow-up work warrants a better understanding of the nature of this off-duty trauma.

Limitations of this survey are the inability to determine causality due to the cross-sectional nature of the study. A second limitation is that these data were obtained through convenience sampling and cannot be determined if these findings are representative of the UN as a whole. Given the large sample size, it is possible that the high rates of individuals screening positive psychiatric disorders and low rates of mental healthcare utilization observed in this study may be found across the UN. Therefore, future research with a representative sample of personnel will be critical for understanding the extent to which these findings are generalizable across the entire organization. Along those lines, there may be differences in the screening rates and factors associated with psychiatric disorders and mental healthcare utilizations across the UN agencies that participated in the survey. However, the data-sharing agreement between the UN and NYU stipulated that the data would not be analyzed or reported at the individual agency level. Another limitation was the total of potential survey participants was estimated, as the exact number of consultants employed at the time of the survey is not included in the published figures on staff totals. It is believed that the presence of consultants did not have a major impact on the findings given that only 1% of the total sample comprised consultants. Additionally, the data in this study may represent non-response bias as it cannot be excluded that missing values were not missing at random. For example, individuals that were younger, not in a relationship, and with less permanent appointments were less likely to answer the clinical measures. Follow up research is needed to better understand whether these patterns are clinically relevant. An alternative explanation may be that those who were newer to the UN with temporary appointments may have been less motivated to complete all of the measures or they may have been more concerned about disclosing information related to psychiatric symptoms. Furthermore, the presence of psychiatric disorders was determined with self-report measures. Previous work has suggested that self-report measures, compared with screening based on clinical interviews, may yield higher rates of those screening positive for mental health disorders due to memory or response biases, and the interpretation of the instructions or items on each self-report measure (e.g. Engelhard *et al*., 2007).

Taken together, the findings of this study strongly suggest that the implementation of a comprehensive mental health strategy is urgently needed throughout the UN. Proactive measures should be taken to actively target those factors at the organizational, environmental, and individual level associated with mental health disorders in this population. UN work is quite diverse in terms of its occupational demands and its environmental and geographic contexts, and interventions may need to be tailored to specific occupational or demographic subgroups. Furthermore, these findings point to the need to better understand barriers to care given to the large mental health services gap observed in this study. Humanitarian aid workers, human rights advocates, and peacekeepers are charged with work such as carrying out research and policy development to the direct provision of basic aid for the world's most vulnerable communities. We recommend that aid and advocacy organizations and agencies partner with mental health researchers to develop policies and programs for addressing psychiatric disorders among people working on many of the world's most urgent issues.

## Footnotes

1 This number is based on the total number of staff employed by these UN agencies published by the UN Office of Human Resource Management and the UNICEF office of Human Resources. These published totals do not include staff with temporary contracts, such as consultants, who would have received an invitation to participate in this survey if they were employed at the time the survey was distributed. However, consultants comprised only 1% small of individuals in the overall sample.

2 The survey that UNICEF distributed was available in three different languages, i.e. English, Spanish and French. Triangulation of the translations was applied to validate the accuracy of translation (e.g. English to Spanish, Spanish to French, French to English).

## Supplementary material

For supplementary material accompanying this paper visit http://dx.doi.org/10.1017/gmh.2019.29. click here to view supplementary material
