# Supplementary material for: Mental health disorders and utilization of mental healthcare services in United Nations personnel
Source: Glob Ment Health (Camb). 2020 Feb 12;7:e5. doi: 10.1017/gmh.2019.29 (PMC7056861; doi:10.1017/gmh.2019.29)
Supplement: Supplementary file 1 [file S2054425119000293sup001.doc]

Mental Health Disorders and

Mental Health Service Utilization in United Nations Personnel

Supplemental Material

PCL, PHQ and GAD Missingness versus Demographic Variables

A combined variable was derived based on PCL, PHQ and GAD missingness. The derived variable “Group” is 1 if subjects fully completed PCL, PHQ and GAD, the derived variable “ Group” is 2 if the subject completed one or two of PCL, PHQ and GAD, the derived variable “Group” is 3 if the subject did not complete any of PCL, PHQ and GAD.

The frequency table between “Group” and gender is shown below. The Chi-square test P value is 0.3329.

| **Table of group by Gender** | | | |
| --- | --- | --- | --- |
| **Group** | **Gender** | | |
| **Frequency Row Percent** | **Female** | **Male** | **Total** |
| **1** | 7348 52.91 | 6540 47.09 | 13888 |
| **2** | 881 54.25 | 743 45.75 | 1624 |
| **3** | 877 51.68 | 820 48.32 | 1697 |
| **Total** | 9106 | 8103 | 17209 |
| **Frequency Missing = 154** | | | |

The frequency table between “group” and age is shown below. The Chi-square test P value is <.0001.

| **Table of group by Age** | | | | | | | |
| --- | --- | --- | --- | --- | --- | --- | --- |
| **group** | **Age** | | | | | | |
| **Frequency Row Percent** | **34 and below** | **35-39** | **40-44** | **45-49** | **50-54** | **55 and above** | **Total** |
| **1** | 2714 19.46 | 2729 19.57 | 2615 18.75 | 2205 15.81 | 1970 14.13 | 1710 12.26 | 13943 |
| **2** | 343 20.98 | 334 20.43 | 288 17.61 | 239 14.62 | 227 13.88 | 204 12.48 | 1635 |
| **3** | 483 28.30 | 313 18.34 | 283 16.58 | 228 13.36 | 221 12.95 | 179 10.49 | 1707 |
| **Total** | 3540 | 3376 | 3186 | 2672 | 2418 | 2093 | 17285 |

The proportion of subjects in each “age” category is tested among groups. The Chi Square test statistic and P values are shown below.

| Age category | Chi Square test statistic | P value |
| --- | --- | --- |
| 34 and below | 73.0897 | <.0001 |
| 35-39 | 2.4029 | 0.3008 |
| 40-44 | 5.5929 | 0.061 |
| 45-49 | 8.0053 | 0.0183 |
| 50-54 | 1.7833 | 0.41 |
| 55 and Above | 4.7475 | 0.0931 |

The proportion of subjects in each “age” category is tested between two groups. The Chi Square test statistic and P values are shown below.

|  | Group 1 vs 2 | | Group 1 vs 3 | | Group 2 vs 3 | |
| --- | --- | --- | --- | --- | --- | --- |
| Age category | Chi Square test statistic | P value | Chi Square test statistic | P value | Chi Square test statistic | P value |
| 34 and below | 2.1256 | 0.1449 | 72.952 | <.0001 | 24.0266 | <.0001 |
| 35-39 | 0.6782 | 0.4102 | 1.4843 | 0.2231 | 2.3408 | 0.126 |
| 40-44 | 1.2549 | 0.2626 | 4.7731 | 0.0289 | 0.6326 | 0.4264 |
| 45-49 | 1.5842 | 0.2082 | 6.9961 | 0.0082 | 1.1046 | 0.2933 |
| 50-54 | 0.0726 | 0.7876 | 1.7655 | 0.1839 | 0.6318 | 0.4267 |
| 55 and Above | 0.0615 | 0.8041 | 4.5298 | 0.0333 | 3.262 | 0.0709 |

The frequency table between “group” and relationship is shown below. The Chi-square test P value is 0.0003.

| **Table of group by Relationship** | | | | |
| --- | --- | --- | --- | --- |
| **group** | **Relationship** | | | |
| **Frequency Row Percent** | **Not in a Relationship** | **In a Partnership Not Recognized by your Organization** | **In a Partnership Recognized by your Organization** | **Total** |
| **1** | 3044 22.02 | 1640 11.86 | 9142 66.12 | 13826 |
| **2** | 369 23.18 | 191 12.00 | 1032 64.82 | 1592 |
| **3** | 438 26.50 | 212 12.83 | 1003 60.68 | 1653 |
| **Total** | 3851 | 2043 | 11177 | 17071 |
| **Frequency Missing = 292** | | | | |

The proportion of subjects in each “relationship” category is tested among groups. The P values are shown below.

| Relationship | Chi Square test statistic | P value |
| --- | --- | --- |
| Not in a Relationship | 17.3546 | 0.0002 |
| In a Partnership Not Recognized by your Organization | 1.3024 | 0.5214 |
| In a Partnership Recognized by your Organization | 19.6867 | <.0001 |

The proportion of subjects in each “relationship” category is tested between two groups. The Chi Square test statistic and P values are shown below.

|  | Group 1 vs 2 | | Group 1 vs 3 | | Group 2 vs 3 | |
| --- | --- | --- | --- | --- | --- | --- |
| Relationship | Chi Square test statistic | P value | Chi Square test statistic | P value | Chi Square test statistic | P value |
| Not in a Relationship | 1.1182 | 0.2903 | 17.0028 | <.0001 | 4.7809 | 0.0288 |
| In a Partnership Not Recognized by your Organization | 0.0251 | 0.874 | 1.3012 | 0.254 | 0.5108 | 0.4748 |
| In a Partnership Recognized by your Organization | 1.0711 | 0.3007 | 19.3768 | <.0001 | 5.9629 | 0.0146 |

The frequency table between “group” and number of children is shown below. The Chi-square test P value is <.0.001.

| **Table of group by Num_child** | | | | | | | | |
| --- | --- | --- | --- | --- | --- | --- | --- | --- |
| **group** | **Num_child** | | | | | | | |
| **Frequency Row Percent** | **0** | **1** | **2** | **3** | **4** | **5** | **More than 5** | **Total** |
| **1** | 5063 45.00 | 1862 16.55 | 2409 21.41 | 1074 9.55 | 439 3.90 | 216 1.92 | 188 1.67 | 11251 |
| **2** | 639 45.03 | 246 17.34 | 294 20.72 | 110 7.75 | 65 4.58 | 27 1.90 | 38 2.68 | 1419 |
| **3** | 736 50.65 | 222 15.28 | 265 18.24 | 101 6.95 | 48 3.30 | 34 2.34 | 47 3.23 | 1453 |
| **Total** | 6438 | 2330 | 2968 | 1285 | 552 | 277 | 273 | 14123 |
| **Frequency Missing = 3240** | | | | | | | | |

The proportion of subjects in each “number of children” category is tested among groups. The P values are shown below.

| Number of Children | Chi Square test statistic | P value |
| --- | --- | --- |
| 0 | 16.7751 | 0.0002 |
| 1 | 2.3133 | 0.3145 |
| 2 | 7.8901 | 0.0193 |
| 3 | 13.9337 | 0.0009 |
| 4 | 3.1245 | 0.2097 |
| 5 | 1.2095 | 0.5462 |
| 6 | 21.2167 | <.0001 |

The proportion of subjects in each “number of children” category is tested between two groups. The Chi Square test statistic and P values are shown below.

|  | Group 1 vs 2 | | Group 1 vs 3 | | Group 2 vs 3 | |
| --- | --- | --- | --- | --- | --- | --- |
| Number of Children | Chi Square test statistic | P value | Chi Square test statistic | P value | Chi Square test statistic | P value |
| 0 | 0.0005 | 0.9822 | 16.5766 | <.0001 | 9.093 | 0.0026 |
| 1 | 0.562 | 0.4535 | 1.5157 | 0.2183 | 2.2279 | 0.1355 |
| 2 | 0.3602 | 0.5484 | 7.7975 | 0.0052 | 2.8183 | 0.0932 |
| 3 | 4.7865 | 0.0287 | 10.3213 | 0.0013 | 0.6763 | 0.4109 |
| 4 | 1.5201 | 0.2176 | 1.2498 | 0.2636 | 3.0982 | 0.0784 |
| 5 | 0.002 | 0.9647 | 1.1775 | 0.2779 | 0.6602 | 0.4165 |
| 6 | 7.2933 | 0.0069 | 17.3308 | <.0001 | 0.7748 | 0.3787 |

The frequency table between group and type of duty is shown below. The Chi-square test P value is 0.1084.

| **Table of group by Type_Duty** | | | |
| --- | --- | --- | --- |
| **Group** | **Type_Duty** | | |
| **Frequency Row Percent** | **Family duty station** | **Non family duty station** | **Total** |
| **1** | 7720 79.62 | 1976 20.38 | 9696 |
| **2** | 959 79.92 | 241 20.08 | 1200 |
| **3** | 621 76.57 | 190 23.43 | 811 |
| **Total** | 9300 | 2407 | 11707 |
| **Frequency Missing = 5656** | | | |

The frequency table between “group” and working years at UN is shown below. The Chi-square test P value is 0.0007.

| **Table of group by Years_UN** | | | | | | |
| --- | --- | --- | --- | --- | --- | --- |
| **group** | **Years_UN** | | | | | |
| **Frequency Row Percent** | **Less than one year** | **1 - 3 Years** | **3 - 5 Years** | **5 - 10 Years** | **More than 10 Years** | **Total** |
| **1** | 1967 14.51 | 2425 17.88 | 1865 13.75 | 3429 25.29 | 3874 28.57 | 13560 |
| **2** | 239 15.41 | 257 16.57 | 209 13.48 | 406 26.18 | 440 28.37 | 1551 |
| **3** | 181 19.72 | 139 15.14 | 115 12.53 | 203 22.11 | 280 30.50 | 918 |
| **Total** | 2387 | 2821 | 2189 | 4038 | 4594 | 16029 |
| **Frequency Missing = 1334** | | | | | | |

The proportion of subjects in each “working years at UN” category is tested among groups. The P values are shown below.

| working years at UN | Chi Square test statistic | P value |
| --- | --- | --- |
| Less than one year | 18.7834 | <.0001 |
| 1 - 3 Years | 5.7119 | 0.0575 |
| 3 - 5 Years | 1.1447 | 0.5642 |
| 5 - 10 Years | 5.4808 | 0.0645 |
| More than 10 Years | 1.6407 | 0.4403 |

The proportion of subjects in each “working years at UN” category is tested between two groups. The Chi Square test statistic and P values are shown below.

|  | Group 1 vs 2 | | Group 1 vs 3 | | Group 2 vs 3 | |
| --- | --- | --- | --- | --- | --- | --- |
| working years at UN | Chi Square test statistic | P value | Chi Square test statistic | P value | Chi Square test statistic | P value |
| Less than one year | 0.9113 | 0.3398 | 18.4772 | <.0001 | 7.5789 | 0.0059 |
| 1 - 3 Years | 1.6449 | 0.1997 | 4.4354 | 0.0352 | 0.8737 | 0.3499 |
| 3 - 5 Years | 0.0912 | 0.7627 | 1.0955 | 0.2953 | 0.4545 | 0.5002 |
| 5 - 10 Years | 0.5809 | 0.446 | 4.61 | 0.0318 | 5.1783 | 0.0229 |
| More than 10 Years | 0.0274 | 0.8684 | 1.5682 | 0.2105 | 1.2648 | 0.2607 |

The frequency table between “group” and contract type is shown below. The Chi-square test P value is <.0001.

| **Table of group by Contract_Type** | | | | | |
| --- | --- | --- | --- | --- | --- |
| **group** | **Contract_Type** | | | | |
| **Frequency Row Percent** | **Permanent or continuous** | **Fixed term** | **Temporary** | **Consultant** | **Total** |
| **1** | 2937 23.95 | 7912 64.51 | 1292 10.53 | 123 1.00 | 12264 |
| **2** | 343 24.78 | 856 61.85 | 170 12.28 | 15 1.08 | 1384 |
| **3** | 222 27.44 | 451 55.75 | 122 15.08 | 14 1.73 | 809 |
| **Total** | 3502 | 9219 | 1584 | 152 | 14457 |
| **Frequency Missing = 2906** | | | | | |

The proportion of subjects in each “contract type” is tested among groups. The P values are shown below.

| Contract Type | Chi Square test statistic | P value |
| --- | --- | --- |
| Permanent or continuous | 5.3062 | 0.0704 |
| Fixed term | 27.6813 | <.0001 |
| Temporary | 18.8332 | <.0001 |
| Consultant | 3.8775 | 0.1439 |

The proportion of subjects in each “contract type” category is tested between two groups. The Chi Square test statistic and P values are shown below.

|  | Group 1 vs 2 | | Group 1 vs 3 | | Group 2 vs 3 | |
| --- | --- | --- | --- | --- | --- | --- |
| Contract Type | Chi Square test statistic | P value | Chi Square test statistic | P value | Chi Square test statistic | P value |
| Permanent or continuous | 0.4751 | 0.4907 | 5.0535 | 0.0246 | 1.886 | 0.1697 |
| Fixed term | 3.8432 | 0.0499 | 25.3043 | <.0001 | 7.8948 | 0.005 |
| Temporary | 3.9745 | 0.0462 | 16.2554 | <.0001 | 3.4608 | 0.0628 |
| Consultant | 0.0813 | 0.7756 | 3.8745 | 0.049 | 1.6364 | 0.2008 |
